# Clinical Factors Associated with Progression and Prolonged Viral Shedding in COVID-19 Patients: A Multicenter Study

**DOI:** 10.14336/AD.2020.0630

**Published:** 2020-10-01

**Authors:** Zhichao Feng, Jennifer Li, Shanhu Yao, Qizhi Yu, Wenming Zhou, Xiaowen Mao, Huiling Li, Wendi Kang, Xin Ouyang, Ji Mei, Qiuhua Zeng, Jincai Liu, Xiaoqian Ma, Pengfei Rong, Wei Wang

**Affiliations:** ^1^Department of Radiology, Third Xiangya Hospital, Central South University, Hunan, China.; ^2^Molecular Imaging Research Center, Central South University, Hunan, China.; ^3^Westmead Clinical School, Faculty of Medicine and Health, University of Sydney, Sydney, Australia.; ^4^Department of Radiology, First Hospital of Changsha, Hunan, China.; ^5^Department of Medical Imaging, First Hospital of Yueyang, Hunan, China.; ^6^Department of Medical Imaging, Central Hospital of Shaoyang, Hunan, China.; ^7^Department of Radiology, Central Hospital of Xiangtan, Hunan, China.; ^8^Department of Radiology, Second Hospital of Changde, Hunan, China.; ^9^Department of Radiology, Central Hospital of Loudi, Hunan, China.; ^10^Department of Radiology, First Affiliated Hospital of University of South China, Hunan, China.

**Keywords:** Viral pneumonia, coronavirus disease 2019, disease progression, viral shedding, risk factors

## Abstract

Coronavirus disease 2019 (COVID-19) is a global pandemic associated with a high mortality. Our study aimed to determine the clinical risk factors associated with disease progression and prolonged viral shedding in patients with COVID-19. Consecutive 564 hospitalized patients with confirmed COVID-19 between January 17, 2020 and February 28, 2020 were included in this multicenter, retrospective study. The effects of clinical factors on disease progression and prolonged viral shedding were analyzed using logistic regression and Cox regression analyses. 69 patients (12.2%) developed severe or critical pneumonia, with a higher incidence in the elderly and in individuals with underlying comorbidities, fever, dyspnea, and laboratory and imaging abnormalities at admission. Multivariate logistic regression analysis indicated that older age (odds ratio [OR], 1.04; 95% confidence interval [CI], 1.02-1.06), hypertension without receiving angiotensinogen converting enzyme inhibitors or angiotensin receptor blockers (ACEI/ARB) therapy (OR, 2.29; 95% CI, 1.14-4.59), and chronic obstructive pulmonary disease (OR, 7.55; 95% CI, 2.44-23.39) were independent risk factors for progression to severe or critical pneumonia. Hypertensive patients without receiving ACEI/ARB therapy showed higher lactate dehydrogenase levels and computed tomography (CT) lung scores at about 3 days after admission than those on ACEI/ARB therapy. Multivariate Cox regression analysis revealed that male gender (hazard ratio [HR], 1.22; 95% CI, 1.02-1.46), receiving lopinavir/ritonavir treatment within 7 days from illness onset (HR, 0.75; 95% CI, 0.63-0.90), and receiving systemic glucocorticoid therapy (HR, 1.79; 95% CI, 1.46-2.21) were independent factors associated with prolonged viral shedding. Our findings presented several potential clinical factors associated with developing severe or critical pneumonia and prolonged viral shedding, which may provide a rationale for clinicians in medical resource allocation and early intervention.

The world has witnessed a rapid escalation of coronavirus disease 2019 (COVID-19), caused by severe acute respiratory syndrome coronavirus 2 (SARS-CoV-2), and it has become a global pandemic since March 2020 [1]. Many patients who are asymptomatic or experience only mild symptoms have a favorable clinical course and recover soon. However, once it progresses to severe COVID-19 or even a critical stage, patients require more intensive medical resource utilization and have a worse prognosis, with a case fatality rate about 20 times higher than that for non-severe patients [2, 3]. To date, there is no specific anti-coronavirus therapy for severe or critical COVID-19, and whether remdesivir is associated with significant clinical benefits for severe COVID-19 still requires further confirmation [4, 5]. A recent report indicated that early triple antiviral therapy may be helpful in alleviating symptoms and shortening the duration of viral shedding in patients with mild-to-moderate COVID-19 [6]. Thus, the key step in reducing the mortality from COVID-19 should be the prevention of progression from non-severe to severe disease stage and the subsequent development of critical illness. Given the fact that the number of confirmed cases is rapidly growing, and effective medical resource is becoming increasingly scarce, there is an urgent need to identify potential high-risk patients to guide reasonable treatment allocation.

Several reports have proposed that the elderly and patients with chronic comorbidities such as hypertension may be at an increased risk of developing severe COVID-19 pneumonia [7-9]. However, it is currently difficult to distinguish between hypertension as an independent risk factor for the development of severe illness in COVID-19 from one that co-varies with other factors such as age and cardiovascular disease [10]. In addition, there has been considerable interest and debate regarding the risk of angiotensinogen converting enzyme inhibitors and angiotensin receptor blockers (ACEI/ARB) therapy in hypertensive patients, presumably through the modulation of the expression of angiotensinogen converting enzyme 2 (ACE2) [9, 11, 12]. Recent studies have shown that among COVID-19 patients with hypertension, ACEI/ARB use was not associated with an increased risk of mortality compared with ACEI/ARB non-use [13, 14]. However, the potential association between ACEI/ARB use and disease progression remains unclear. In addition, the viral ribonucleic acid (RNA) excretion pattern in respiratory specimens during the treatment of COVID-19 has been analyzed in a limited number of studies, and the factors associated with prolonged viral shedding in COVID-19 patients with different disease severities in large sample population deserve further investigation [15, 16].

Therefore, we conducted this multicenter, retrospective study to determine the clinical risk factors associated with disease progression and prolonged viral shedding in patients with COVID-19 in Hunan Province, China.

## MATERIALS AND METHODS

### Study population

The Institutional Review Board of the Third Xiangya Hospital of Central South University approved our study and waived the need for informed consent because of the retrospective nature of this study. All consecutive adult patients with confirmed COVID-19 who were treated at the Third Xiangya Hospital of Central South University, Changsha Public Health Treatment Center, First Hospital of Yueyang, Junshan People’s Hospital of Yueyang, Central Hospital of Shaoyang, Central Hospital of Xiangtan, Second Hospital of Changde, Central Hospital of Loudi, and First Affiliated Hospital of University of South China between January 17, 2020 and February 28, 2020 were enrolled. Patients without available clinical or imaging data were excluded. The diagnosis of COVID-19 was established on the basis of the World Health Organization interim guidelines, and a confirmed case was defined as positive on the basis of the presence of SARS-CoV-2 RNA on high-throughput sequencing or real-time reverse transcription-polymerase chain reaction (RT-PCR) assay in nasal and pharyngeal swab specimens.

### Collection of clinical and imaging data

Data on demographic, epidemiological history, chronic comorbidities, clinical symptoms, and laboratory findings were obtained at admission from electronic medical records. Medication history among individuals with hypertension was recorded, including treatment with ACEIs or ARBs, calcium antagonists, β-blockers, and diuretic agents. Body mass index (BMI) was calculated as weight divided by height squared, and obesity was defined as BMI ≥ 28 kg/m^2^ according to the Chinese-specific cut-offs for general adiposity [17]. All laboratory tests were performed according to the clinical care need of the patient during hospitalization, including complete blood count, assessment of liver and renal function, coagulation testing, and measures of electrolyte, creatine kinase, lactate dehydrogenase (LDH), procalcitonin, and C-reactive protein levels. Respiratory tract specimens were collected daily from patients during the course of hospitalization and tested for SARS-CoV-2 RNA. The treatment protocol for each patient during hospitalization was also recorded, and the exposure of lopinavir/ritonavir was defined as having received at least one dosage of lopinavir/ritonavir treatment. CT images were acquired at admission and at intervals of 3-5 days during hospitalization and were reviewed independently by two experienced radiologists. Typical CT findings for COVID-19 were defined as peripherally distributed multifocal ground-glass opacities (GGOs) with patchy consolidations and a predilection for involvement of the posterior part or lower lobe of the lung [18]. Each of the five lung lobes was reviewed for GGO and consolidation. The extent of the lesions within each lung lobe was semi-quantitatively evaluated by scoring from 0 to 5 based on the degree of involvement, as follows: score 0, no involvement; score 1, ≤ 5% involvement; score 2, 6%-25% involvement; score 3, 26%-50% involvement; score 4, 51%-75% involvement; and score 5, >75% involvement. The total score was calculated by summing the scores of all five lobes to provide a lung CT score ranging from 0 to 25 [19]. The mean values of lung CT scores evaluated by the two radiologists were used for analysis.

**Table 1:** Clinical and laboratory characteristics of 564 COVID-19 patients according to disease severity.

Characteristics	Total (n =564)	Severe or critical (n = 69)	Mild or moderate (n = 495)	*P*
Age (years)	47 (36-58)	59 (51-66)	45 (35-56)	< 0.001
Male gender	284 (50.4%)	39 (56.5%)	245 (49.5%)	0.274
Smoking history	47 (8.3%)	9 (13.0%)	38 (7.7%)	0.131
Comorbidity				
Any	132 (23.4%)	37 (53.6%)	95 (19.2%)	< 0.001
Hypertension	82 (14.5%)	21 (30.4%)	61 (12.3%)	< 0.001
Diabetes	45 (8.0%)	12 (17.4%)	33 (6.7%)	0.002
Cardiovascular disease	22 (3.9%)	6 (8.7%)	16 (3.2%)	0.062
COPD	16 (2.8%)	9 (13.0%)	7 (1.4%)	< 0.001
Cerebrovascular disease	5 (0.9%)	0 (0)	5 (1.0%)	0.878
Chronic renal disease	3 (0.5%)	2 (2.9%)	1 (0.2%)	0.045
Hepatitis B/C infection	9 (1.6%)	3 (4.4%)	6 (1.2%)	0.151
Malignancy	4 (0.7%)	1 (1.5%)	3 (0.6%)	0.987
Immunodeficiency	1 (0.2%)	0 (0)	1 (0.2%)	0.249
Obesity	49 (8.7%)	10 (14.5%)	39 (7.9%)	0.068
Symptoms or signs				
Fever	357 (63.3%)	58 (84.1%)	299 (60.4%)	< 0.001
Cough	323 (57.2%)	37 (53.6%)	286 (57.8%)	0.513
Fatigue	87 (15.4%)	8 (11.6%)	79 (16.0%)	0.347
Myalgia	25 (4.4%)	3 (4.4%)	22 (4.4%)	0.783
Sputum production	51 (9.0%)	6 (8.7%)	45 (9.1%)	0.915
Anorexia	26 (4.6%)	6 (8.7%)	20 (4.0%)	0.084
Diarrhea	18 (3.2%)	4 (5.8%)	14 (2.8%)	0.343
Dyspnea	26 (4.6%)	7 (10.1%)	19 (3.8%)	0.019
Hemoglobin, g/L	131 (121-143)	131 (119-142)	132 (121-144)	0.258
Platelet count, ×10^9^/L	182 (143-236)	163 (138-234)	183 (144-237)	0.390
Blood leukocyte count, ×10^9^/L	4.8 (3.7-6.1)	5.6 (3.9-8.3)	4.7 (3.6-5.9)	0.004
Neutrophil count, × 10?/L	3.0 (2.2-4.2)	4.4 (2.7-6.8)	2.9 (2.2-3.9)	< 0.001
Lymphocyte count, × 10?/L	1.1 (0.8-1.5)	0.7 (0.5-1.1)	1.2 (0.9-1.6)	< 0.001
Alanine aminotransferase, U/L	20.3 (15.0-30.4)	24.7 (16.8-41.0)	20.0 (15.0-29.5)	0.011
Aspartate aminotransferase, U/L	24.3 (19.5-31.5)	35.0 (25.6-47.0)	23.8 (19.0-29.0)	< 0.001
Total bilirubin, μmol/L	11.9 (8.7-17.6)	11.8 (9.0-17.4)	11.9 (8.7-17.6)	0.887
Albumin, g/L	39.0 (35.7-42.4)	35.4 (31.7-38.3)	39.4 (36.4-42.7)	< 0.001
Blood urea nitrogen, mmol/L	4.0 (3.2-5.0)	4.9 (3.7-6.2)	3.9 (3.1-4.8)	< 0.001
Creatinine, μmol/L	59.0 (46.8-74.0)	65.1 (53.0-84.0)	58.2 (46.2-72.9)	0.002
Sodium, mmol/L	137.6 (135.4-140.1)	135.4 (133.3-137.2)	138.0 (135.9-140.3)	< 0.001
Potassium, mmol/L	4.0 (3.6-4.3)	3.9 (3.5-4.3)	4.0 (3.6-4.3)	0.493
Creatine kinase, U/L	70.8 (48.0-112.8)	81.7 (48.0-181.2)	70.0 (48.0-109.3)	0.092
Lactose dehydrogenase, U/L	189.0 (152.0-244.0)	304.4 (236.8-378.5)	183.6 (148.3-230.3)	< 0.001
D-dimer ≥ 0.05 mg/L	269 (47.7%)	44 (63.8%)	225 (45.5%)	0.004
Procalcitonin ≥ 0.05 ng/L	207 (36.7%)	38 (55.1%)	169 (34.1%)	0.001
C-reactive protein ≥ 10 mg/L	286 (50.7%)	61 (88.4%)	225 (45.5%)	< 0.001
Abnormal CT findings	516 (91.5%)	68 (98.6%)	448 (90.5%)	0.025
Lung CT score	6 (4-11)	15 (10-21)	6 (3-9)	< 0.001

Abbreviations: COPD, chronic obstructive pulmonary disease; COVID-19; 2019 coronavirus disease; CT, computed tomography.

The disease severity of COVID-19 was defined as follows: (1) mild type, with fever or respiratory tract symptoms but without radiological pneumonia; (2) moderate type, with fever, respiratory tract symptoms, and radiological evidence of pneumonia; (3) severe type, with one of the following: a) respiratory distress (respiratory rate ≥ 30 beats/min), b) hypoxia (oxygen saturation ≤ 93% in the resting state), or c) hypoxemia (arterial blood oxygen partial pressure/oxygen concentration ≤ 300mmHg); (4) critical type, with one of the following: a) respiratory failure requiring mechanical ventilation, b) shock, or c) intensive care unit (ICU) admission because of combined other organ failure.

Duration of viral shedding after illness onset was considered as the number of days from symptom onset to persistent negative results on respiratory tract viral RT-PCR testing. All samples from the same patient were tested until two consecutive samples showed negative results, with the first negative result defining the duration of shedding.

### Statistical Analysis

Continuous variables are presented as the median and interquartile range (IQR), and categorical variables are presented as frequency and percentage. Differences between groups were analyzed using Student’s t-test or the Mann-Whitney U test for quantitative variables according to the normal distribution and the Chi-square test or Fisher’s exact test for categorical variables. Univariate and multivariate logistic regressions with backward stepwise selection on the basis of the likelihood ratio were used to identify the risk factors for the development of severe or critical pneumonia, and the corresponding odds ratios (ORs) and 95% confidence intervals (CIs) were determined. The cumulative percentage of patients with positive SARS-CoV-2 RNA was calculated using Kaplan Meier curves, and comparisons between groups were performed using the log-rank test. The Cox proportional hazard model was adopted for multivariate analyses of potential risk factors associated with the discontinuation of viral shedding, and the hazard ratio (HR) and 95% CI were calculated. The analyses regarding different factors were based on non-missing data, and missing data were not imputed. All statistical analyses were performed using SPSS statistics software (version 22.0, IBM SPSS Inc., Chicago, IL, USA). A two-sided *P* value of less than 0.05 was considered statistically significant.

**Figure 1.**
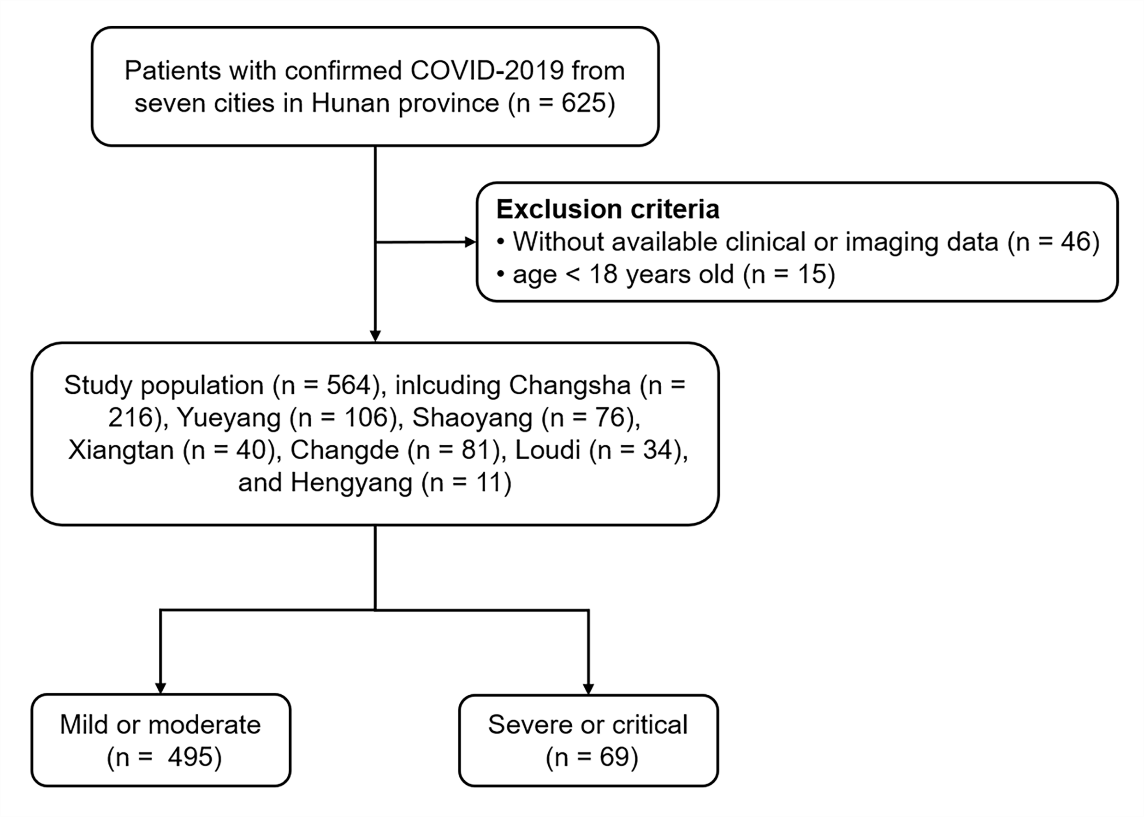
The flow chart of the study design.

## RESULTS

### Patient Characteristics

**Figure 2.**
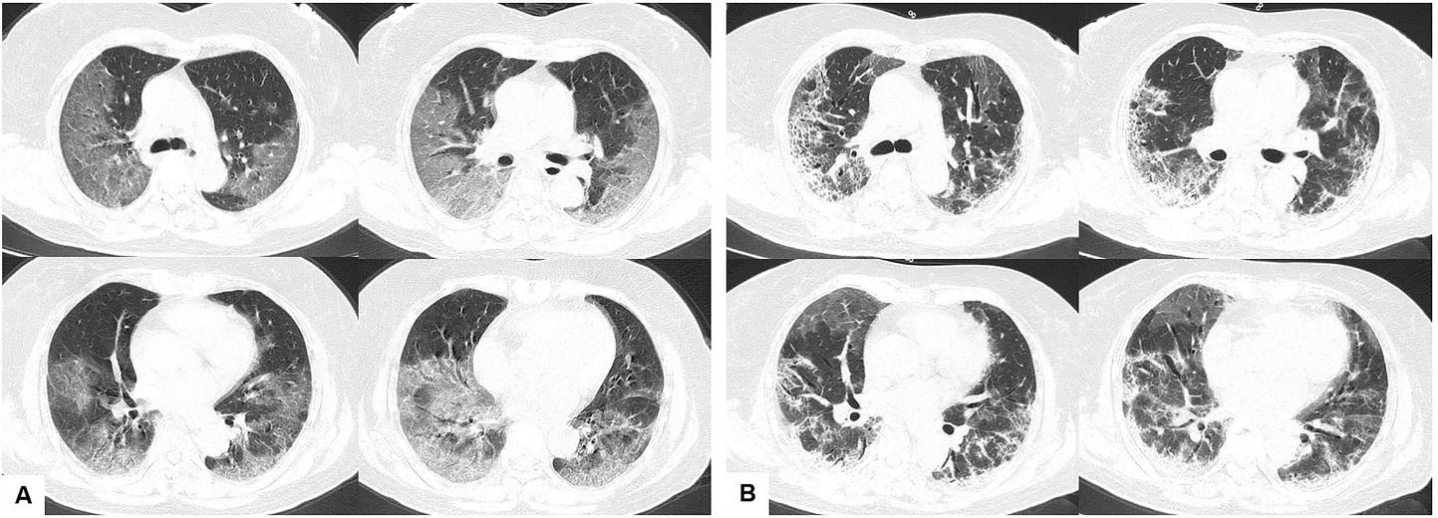
Chest CT images of a 63-year-old female patient with severe COVID-19 pneumonia. (A) Chest CT images obtained on January 24, 2020, show bilateral GGO with lung CT score of 15 on day 10 after symptom onset. (B) CT images obtained on February 2, 2020, show partial absorption and local progression of GGO with patchy of consolidation after treatment. COVID-19; 2019 coronavirus disease; CT, computed tomography; GGO, ground glass opacity.

The study population included 564 adult patients with confirmed COVID-19 from nine hospitals in seven cities (Fig. 1). The clinical characteristics of these patients at admission are presented in Table 1. The median age of patients was 47 years (IQR, 36-58 years; range, 19-84 years), and 284 (50.4%) were men. A total of 132 (23.4%) patients had comorbidities, including hypertension (n = 82 [14.5%]), diabetes (n = 45 [8.0%]), cardiovascular disease (n = 22 [3.9%]), and chronic obstructive pulmonary disease (COPD, n = 16 [2.8%]). There were 49 (8.7%) patients with obesity. The most common self-reported symptoms at onset of illness were fever (n = 357 [63.3%]), cough (n = 323 [57.2%]), fatigue (n = 87 [15.4%]), sputum production (n = 51 [9.0%]), anorexia (n = 26 [4.6%]), and dyspnea (n = 26 [4.6%]). A total of 516 (91.5%) patients had typical abnormal findings on chest CT (Fig. 2), with a median lung CT score of 6 (IQR, 4-11; range, 0-25). Sixty-nine of the 564 (12.2%) patients were identified as having severe or critical pneumonia. As summarized in Table 1, these patients were older (*P* < 0.001) and more likely to have preexisting comorbidities, including hypertension, diabetes, COPD, and chronic renal disease (all*P* < 0.05), when compared with patients with non-severe disease. Patients who were obese showed a trend towards to develop severe or critical pneumonia though without statistically significance (*P* = 0.068). The clinical symptoms at illness onset also differed, with those suffering from severe or critical disease more likely to present with fever and dyspnea (both*P* < 0.05). Furthermore, patients with severe or critical pneumonia had elevated liver enzyme levels (aspartate aminotransferase, *P* < 0.001), renal dysfunction (blood urea nitrogen, *P* < 0.001), abnormal coagulation function (D-dimer level ≥ 0.05 mg/L, *P* = 0.004), increased inflammation-related parameters (LDH, *P* < 0.001), and a worse lung CT score (*P* < 0.001). However, lymphocyte count (*P* < 0.001) was significantly lower in patients with severe or critical pneumonia.

**Table 2:** Clinical risk factors associated with development of severe or critical illnesses.

Variables	Univariate OR (95% CI)	*P* value	Multivariate OR (95% CI)	*P* value
Age	1.06 (1.04-1.08)	< 0.001	1.04 (1.02-1.07)	< 0.001
Male gender	1.33 (0.80-2.20)	0.275		
Smoking history	1.80 (0.83-3.92)	0.136		
Hypertension	3.11 (1.75-5.55)	< 0.001		0.064
Diabetes	2.95 (1.44-6.03)	0.003		0.159
Cardiovascular disease	2.85 (1.08-7.56)	0.035		0.807
COPD	13.81 (4.85-39.38)	< 0.001	7.53 (2.44-23.26)	< 0.001
Obesity	1.98 (0.94-4.18)	0.072		0.060
Age	1.06 (1.04-1.08)	< 0.001	1.04 (1.02-1.06)	< 0.001
Male gender	1.33 (0.80-2.20)	0.275		
Smoking history	1.80 (0.83-3.92)	0.136		
Hypertension		< 0.001		0.046
Non-hypertensive	Reference	1	Reference	1
Hypertensive on AECI/ARB therapy	0.60 (0.08-4.66)	0.628	0.51 (0.06-3.99)	0.517
Hypertensive on other therapy	3.93 (2.15-7.19)	< 0.001	2.29 (1.14-4.59)	0.020
Diabetes	2.95 (1.44-6.03)	0.003		0.210
Cardiovascular disease	2.85 (1.08-7.56)	0.035		0.944
COPD	13.81 (4.85-39.38)	< 0.001	7.55 (2.44-23.39)	< 0.001
Obesity	1.98 (0.94-4.18)	0.072		0.086

Abbreviations: ACEI, angiotensin-converting enzyme inhibitors; ARB, angiotensin-receptor blockers; CI, confidence interval; COPD, chronic obstructive pulmonary disease; OR, odds ratio.

### Factors associated with the development of severe or critical COVID-19

To investigate the clinical risk factors for the development of severe or critical pneumonia in COVID-19 patients, univariate and multivariate logistic regression analyses were performed (Table 2). Univariate analysis identified age, hypertension, diabetes, cardiovascular disease, and COPD to be associated with the development of severe or critical pneumonia (all *P* < 0.05), while only older age (OR, 1.04; 95% CI, 1.02-1.07; *P* < 0.001) and COPD (OR, 7.53; 95% CI, 2.44-23.26; *P* < 0.001) were identified as independent risk factors in multivariate analysis. Furthermore, after stratification according to whether patients with hypertension were receiving ACEI/ARB therapy or not, the presence of hypertension without receiving ACEI/ARB therapy became independently associated with the development of severe or critical pneumonia (OR, 2.29; 95% CI, 1.14-4.59; *P* = 0.020). Compared with patients without hypertension, hypertension on ACEI/ARB therapy was not associated with the development of severe or critical pneumonia (OR, 0.51; 95% CI, 0.06-3.99; *P* = 0.517). Besides, subgroup analyses according to age or gender showed that hypertension without receiving ACEI/ARB therapy and COPD remained significantly associated with disease progression only among the older patients (age ≥ 50 years), and only older age was significantly associated with disease progression for both male and female patients (Supplementary Table 1-2).

### ACEI/ARB therapy in patients with hypertension

**Figure 3.**
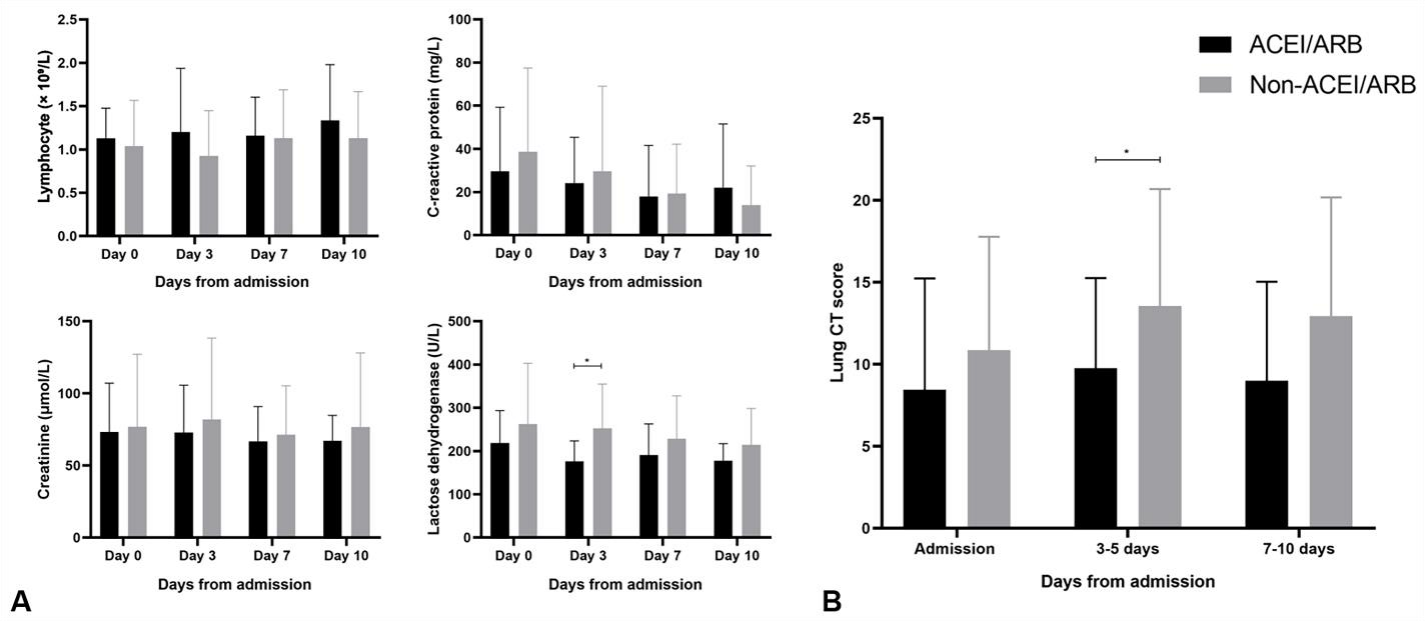
Dynamic profile of laboratory and imaging findings between hypertensive patients on ACEI/ARB therapy and those on non-ACEI/ARB therapy. Timeline bar graphs illustrate the laboratory (A) and imaging (B) parameters in 65 patients based on the days after admission. ^*^ *P* < .05 for patients on ACEI/ARB therapy vs patients on non-ACEI/ARB therapy.

In our cohort, 82 individuals were recorded as having hypertension and only 16 were on ACEI/ARB therapy (including 6 patients receiving ARB monotherapy, 9 receiving combined ARB and calcium antagonist, and 1 receiving AECI monotherapy). There were 49 patients on non-ACEI/ARB antihypertensive therapy (including 39 patients receiving calcium antagonist monotherapy, 3 receiving β-blocker monotherapy, 2 receiving diuretic monotherapy, and 5 receiving combined calcium antagonist and β-blocker therapy), and all were maintained on their usual antihypertensive regimen during hospitalization. The remaining 17 patients were not on antihypertensive medications or had unavailable data and received amlodipine or nitrendipine to control blood pressure during hospitalization as necessary. As summarized in Table 3, hypertensive patients on ACEI/ARB therapy had similar baseline clinical characteristics and laboratory findings as those on non-ACEI/ARB therapy. However, in contrast with those on ACEI/ARB therapy, patients who were not receiving ACEI/ARB therapy were more likely to develop severe or critical pneumonia (*P* = 0.037). Laboratory and imaging data from 65 patients with hypertension during hospitalization were analyzed (Figure 3), showing that, at about 3 days after admission, LDH levels and CT lung scores were higher in patients who were not receiving ACEI/ARB therapy than in those on ACEI/ARB therapy.

**Table 3:** Comparison between hypertensive patients on ACEI/ARB therapy and on non-ACEI/ARB therapy (n = 65).

Characteristics	ACEI/ARB (n = 16)	Non-ACEI/ARB (n = 49)	*P*
Age (years)	57 (51-65)	63 (53-69)	0.106
Male gender	10 (62.5%)	23 (46.9%)	0.280
Smoking history	2 (12.5%)	5 (10.2%)	0.836
Comorbidity			
Diabetes	2 (12.5%)	18 (36.7%)	0.131
Cardiovascular disease	0 (0)	8 (16.3%)	0.198
COPD	0 (0)	1 (2.0%)	0.553
Cerebrovascular disease	0 (0)	3 (6.1%)	0.311
Chronic renal disease	1 (6.3%)	1 (2.0%)	0.435
Hepatitis B infection	0 (0)	2 (4.1%)	0.412
Obesity	1 (6.3%)	10 (20.4%)	0.354
Medication history			
Aspirin	0 (0)	3 (6.1%)	0.311
Statins	1 (6.3%)	2 (4.1%)	0.743
Metformin	1 (6.3%)	8 (16.3%)	0.551
Other oral hypoglycemic agents	1 (6.3%)	10 (20.4%)	0.354
Insulin	0 (0)	2 (4.1%)	0.412
Systolic pressure, mmHg	132 (120-137)	136 (120-146)	0.107
Diastolic pressure, mmHg	80 (71-89)	80 (78-87)	0.547
Hemoglobin, g/L	133 (125-150)	133 (118-138)	0.060
Platelet count, ×10^9^/L	181 (135-241)	182 (147-239)	0.837
Blood leukocyte count, ×10^9^/L	5.5 (4.6-6.5)	5.2 (3.9-7.0)	0.698
Neutrophil count, × 10?/L	3.7 (3.1-4.4)	3.7 (2.4-4.8)	0.733
Lymphocyte count, × 10?/L	1.1 (0.9-1.4)	1.0 (0.7-1. 5)	0.322
Alanine aminotransferase, U/L	28.9 (16.2-40.7)	23.0 (17.2-31.5)	0.590
Aspartate aminotransferase, U/L	25.0 (20.0-30.9)	26.3 (20.0-36.3)	0.800
Total bilirubin, μmol/L	12.6 (10.3-19.9)	12.5 (9.5-19.0)	0.634
Albumin, g/L	38.4 (34.6-44.5)	37.0 (32.2-40.4)	0.185
Blood urea nitrogen, mmol/L	4.8 (4.1-5.8)	4.5 (3.7-6.5)	0.611
Creatinine, μmol/L	64.7 (44.9-82.1)	63.3 (51.2-78.7)	0.877
Sodium, mmol/L	136.3 (134.7-137.2)	137.5 (134.6-140.6)	0.252
Potassium, mmol/L	4.0 (3.6-4.4)	3.9 (3.5-4.2)	0.438
Creatine kinase, U/L	118.5 (64.6-187.3)	64.5 (46.5-147.2)	0.083
Lactose dehydrogenase, U/L	184.0 (157.1-281.7)	238.0 (164.1-315.2)	0.301
D-dimer ≥ 0.05 mg/L	10 (62.5%)	27 (55.1%)	0.604
Procalcitonin ≥ 0.05 ng/L	6 (37.5%)	27 (55.1%)	0.221
C-reactive protein ≥ 10 mg/L	11 (68.8%)	41 (83.7%)	0.195
Abnormal CT findings	15 (93.8%)	46 (93.9%)	0.985
Lung CT score	7 (3-14)	10 (5-16)	0.258
Combined treatments after admission		
Antiviral therapy	15 (93.8%)	45 (91.8%)	0.803
Interferon α	8 (50.0%)	35 (71.4%)	0.116
Intravenous antibiotics	12 (75.0%)	34 (69.4%)	0.668
Severe or critical illnesses	1 (6.3%)	16 (32.7%)	0.037

Abbreviations: ACEI, angiotensin-converting enzyme inhibitors; ARB, angiotensin-receptor blockers; COPD, chronic obstructive pulmonary disease; CT computed tomography.

### Factors associated with prolonged viral shedding

**Figure 4.**
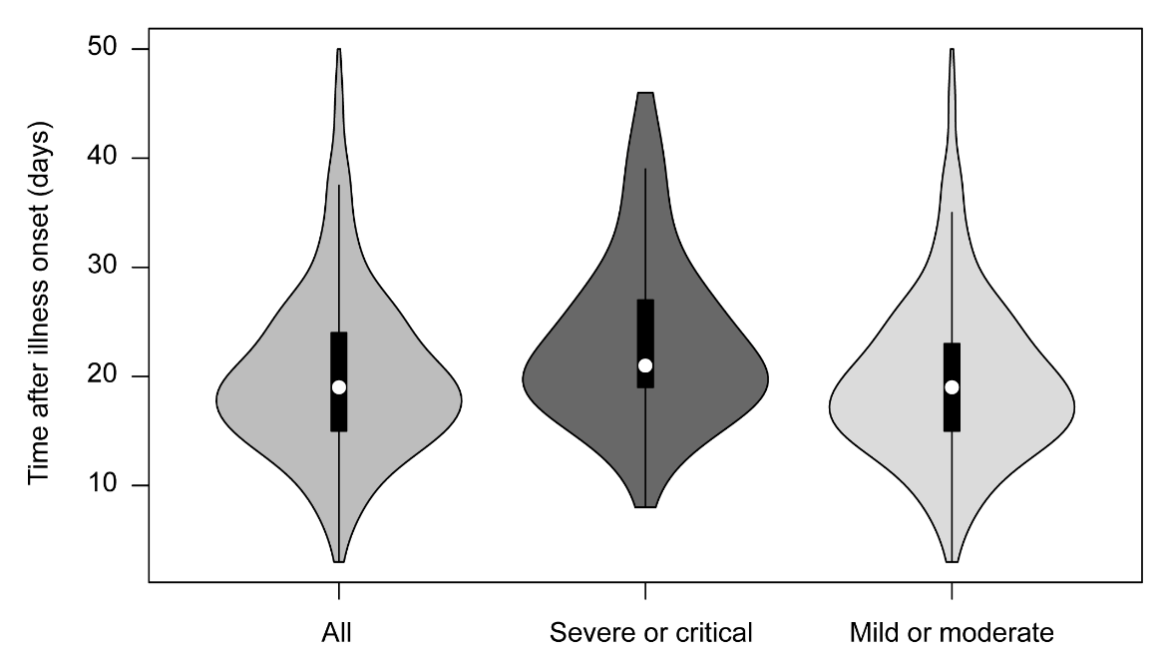
Duration of SARS-CoV-2 viral shedding by disease severity. The white dots represent medians, the black bars represent interquartile ranges, and the thin black lines represent upper and lower adjacent values. SARS-CoV-2, severe acute respiratory syndrome coronavirus 2.

The median duration of viral shedding of all patients, patients with severe or critical disease, and patients with mild or moderate disease were 19 days, 21 days, and 19 days, respectively (Fig. 4). Univariate Cox regression analysis showed that older age (*P* = 0.032), any comorbidity (*P*= 0.028), severe or critical illnesses (*P* = 0.002), receiving lopinavir/ritonavir treatment within 7 days from illness onset (*P* = 0.013), receiving systemic glucocorticoid therapy (*P*<0.001), and invasive mechanical ventilation (*P* = 0.011) were found to be significantly associated with the duration of viral shedding in patients with COVID-19 (Table 4). In the multivariate analysis, male gender (HR, 1.22; 95% CI, 1.02-1.46), receiving lopinavir/ritonavir treatment within 7 days from illness onset (HR, 0.75; 95% CI, 0.63-0.90), and receiving systemic glucocorticoid therapy (HR, 1.79; 95% CI, 1.46-2.21) were identified to be independently associated with prolonged viral shedding.

**Table 4:** Clinical risk factors associated with prolonged viral shedding.

Variables	Univariate HR (95% CI)	*P* value	Multivariate HR (95% CI)	*P* value
Age	1.01 (1.00-1.01)	0.032		0.770
Male gender	1.15 (0.97-1.39)	0.102	1.22 (1.02-1.46)	0.030
Smoking history	0.83 (0.60-1.12)	0.227		
Any comorbidity	1.25 (1.02-1.54)	0.028		0.202
Hypertension		0.106		0.502
Non-hypertensive	Reference	1		1
Hypertensive on AECI/ARB therapy	0.63 (0.37-1.08)	0.095		0.383
Hypertensive on other therapy	0.82 (0.62-1.08)	0.159		0.485
Diabetes	0.99 (0.72-1.39)	0.976		
Cardiovascular disease	1.29 (0.82-2.02)	0.268		
COPD	0.74 (0.44-1.24)	0.248		
Obesity	1.23 (0.90-1.69)	0.197		
Severe or critical illnesses	1.52 (1.16-1.96)	0.002		0.340
Lopinavir/ritonavir treatment within 7 days from illness onset	0.80 (0.67-0.95)	0.013	0.75 (0.63-0.90)	0.002
Systematic glucocorticoid therapy	1.67 (1.35-2.04)	< 0.001	1.79 (1.46-2.21)	< 0.001
Invasive mechanical ventilation	1.96 (1.16-3.23)	0.011		0.877

Abbreviations: ACEI, angiotensin-converting enzyme inhibitors; ARB, angiotensin-receptor blockers; CI, confidence interval; COPD, chronic obstructive pulmonary disease; HR, hazard ratio.

Kaplan Meier curves between male and female patients (log-rank *P* = 0.093), between patients who received lopinavir/ritonavir treatment within 7 days from illness onset and those who did not (log-rank *P* = 0.009), and between patients who received systematic glucocorticoid therapy and those who did not (log-rank *P* < 0.001) are shown in Figure 4. Further stratification analysis showed that male gender (HR, 1.21; 95% CI, 1.00-1.47), receiving lopinavir/ritonavir treatment within 7 days from illness onset (HR, 0.76; 95% CI, 0.62-0.92), and receiving systemic glucocorticoid therapy (HR, 1.69; 95% CI, 1.32-2.17) remained independently associated with prolonged viral shedding only in patients with mild or moderate COVID-19, while among those with severe or critical COVID-19, older age (HR, 1.04; 95% CI, 1.02-1.06) and receiving systemic glucocorticoid therapy (HR, 1.85; 95% CI, 1.05-3.26) were confirmed to be independently associated with prolonged viral shedding (Table 5).

**Figure 5.**
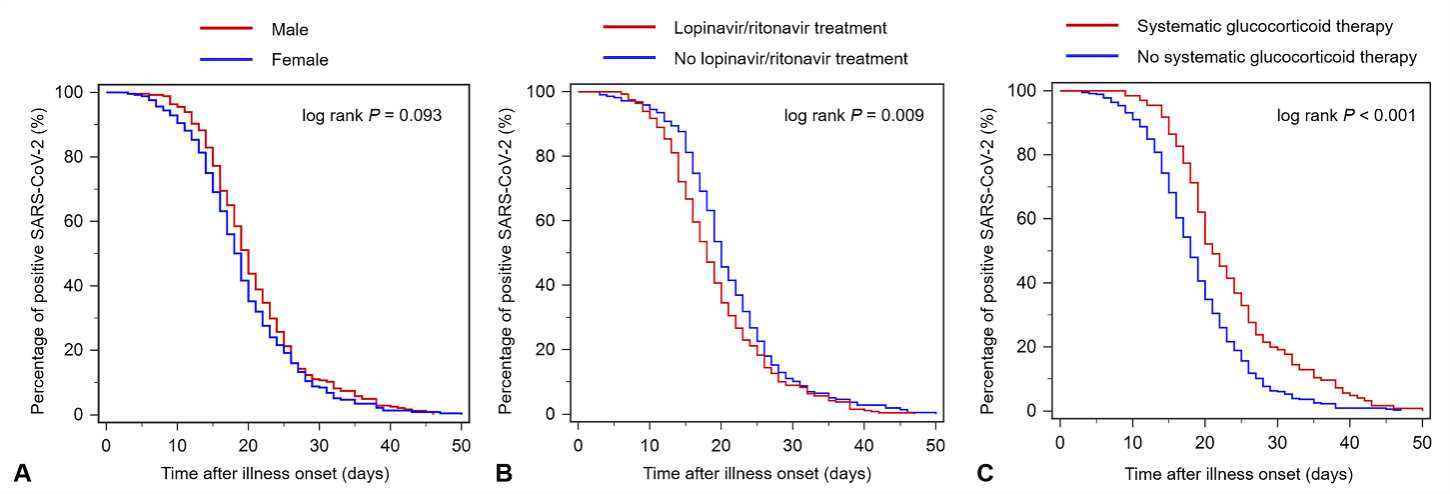
Cumulative percentage of patients with positive SARS-CoV-2 RNA by time after illness onset. (A) Kaplan-Meier curves between male and female patients. (B) Kaplan-Meier curves between patients who received lopinavir/ritonavir treatment within 7 days from illness onset and those who did not. (C) Kaplan-Meier curves between patients who received systematic glucocorticoid therapy and those who did not. RNA, ribonucleic acid; SARS-CoV-2, severe acute respiratory syndrome coronavirus 2.

## DISCUSSION

In this study, we described the clinical risk factors associated with disease progression and prolonged viral shedding in patients with COVID-19. Our results showed that older age, hypertension without receiving ACEI/ARB therapy, and COPD were independent risk factors for progression to severe or critical pneumonia. In addition, male gender, receiving lopinavir/ritonavir treatment within 7 days from illness onset, and receiving systemic glucocorticoid therapy were found to be associated with prolonged viral shedding in COVID-19 patients.

**Table 5:** Clinical risk factors associated with prolonged viral shedding according to disease severity.

Variables	Severe or critical		Mild or moderate	
	Multivariate HR (95% CI)	*P* value	Multivariate HR (95% CI)	*P* value
Age	1.04 (1.02-1.06)	0.001		0.362
Male gender		0.481	1.21 (1.00-1.47)	0.049
Smoking history		0.445		0.473
Any comorbidity		0.924		0.506
Hypertension		0.535		0.577
Non-hypertensive		1		1
Hypertensive on AECI/ARB therapy		0.955		0.299
Hypertensive on other therapy		0.266		0.824
Diabetes		0.888		0.663
Cardiovascular disease		0.121		0.292
COPD		0.499		0.894
Obesity		0.813		0.174
Lopinavir/ritonavir treatment within 7 days from illness onset		0.414	0.76 (0.62-0.92)	0.005
Systematic glucocorticoid therapy	1.85 (1.05-3.26)	0.034	1.69 (1.32-2.17)	< 0.001
Invasive mechanical ventilation		0.409		—

Abbreviations: ACEI, angiotensin-converting enzyme inhibitors; ARB, angiotensin-receptor blockers; CI, confidence interval; COPD, chronic obstructive pulmonary disease; HR, hazard ratio.

Consistent with the clinical experience around the world, our cohort study also confirmed that older age was independently associated with disease progression in patients with COVID-19. The association between age and disease progression may be attributed to the accumulation of medical comorbidities with age or a decline in immune function over time [20]. Previous data were not adjusted for confounding factors of age and other comorbidities to definitively show whether hypertension was independently associated with worse outcomes [7, 8]. Given the close association between age and hypertension, our study adjusted for age and revealed that hypertension was not an independent risk factor for disease progression. Consistent with a recent report, COPD was also found to predispose COVID-19 patients to adverse clinical outcomes, which may be explained by the insufficiency of pulmonary function reserve capacity [21]. Besides, our results showed patients who were obese had a trend towards developing severe or critical pneumonia, but obesity was not confirmed as an independent risk factor associated with progression by multivariate analysis. Though these findings were slightly inconsistent with previous reports, obese patients with COVID-19 should be paid more attention in aggressive management to prevent progression [22, 23].

We further performed stratification analysis for hypertension according to whether receiving ACEI/ARB antihypertensive therapy and revealed that among patients with hypertension, only those who were not receiving ACEI/ARB therapy had a higher risk of developing severe or critical pneumonia, although we acknowledged that the analysis was conducted within a limited sample size - with only 16 of 82 patients on ACEI/ARB therapy. The dynamic profile of laboratory and imaging findings showed that LDH levels and CT lung scores were higher among patients without receiving ACEI/ARB therapy than among those on ACEI/ARB therapy, at about 3 days after admission. Damage to the liver, kidney, or lung in severe or critical COVID-19 attacks may contribute to cellular death and LDH leakage, consequently increasing serum LDH levels, while CT lung score is a semi-quantitative index associated with lung involvement [20, 24]. These dynamic results indicated that hypertensive patients without receiving ACEI/ARB therapy had more severe multiple organ damage peaks, which made them more susceptible to severe or critical pneumonia.

COVID-19 patients with hypertension may have worse outcomes due to decreased ACE2 levels. ACE2 is highly expressed in both the lung and myocardium and converts angiotensin II (AII) into angiotensin 1-7, which can downregulate ACE and directly cause vasodilatation [25-27]. It has been proposed that the binding of the SARS-CoV-2 spike glycoprotein (S-protein) to the functional receptor ACE2 results in proteolytic cleavage by type II transmembrane serine proteases (TMPRSS2) [28-30]. Downregulation of ACE2 levels has also been shown in hypertensive animal models, although there has been limited success in translational confirmation in human studies [31, 32]. None the less, this may be exacerbated by the further reduction in available ACE2 following coronavirus infection. Both SARS-CoV and SARS-CoV-2 can gain entry into the host cell through binding of the S-protein to the membrane-bound ACE2 aminopeptidase, which is then cleaved by serine proteases to allow for membrane fusion and invasion [30, 33]. Following infection, there is a reduction in pulmonary ACE2, either through internalization with viral entry and/or downregulation of ACE2 enzyme during this process. Hence, this may exacerbate the lower baseline ACE2 function in hypertensive patients, leading to the accumulation of AII and reduced angiotensin 1-7, and subsequently worse immune-related cytolysis and lung injury, which was validated by the linear association between elevated plasma AII levels and viral load or lung injury in COVID-19 patients [34].

The use of ACEI or ARB likely promotes feedback upregulation of ACE2 expression in hypertension, although animal studies have found either no effect or even downregulation of ACE2 levels in the context of cardiovascular disease with ACEI/ARB therapy [27, 35-37]. Nonetheless, this may explain our finding that ACEI/ARB therapy may be protective against severe infection. Further evidence that would support this hypothesis comes from earlier studies in which ACEI/statins were protective against severe pneumonia, recombinant ACE2 protected mice from lung injury following SARS-CoV infection, and ARBs reduced acute lung injury in rats injected with SARS-CoV S-protein [38, 39].

As can be seen from our results, patients who had received systemic glucocorticoid therapy had a longer duration of viral RNA shedding than did those who did not. Glucocorticoid treatment might efficiently inhibit T-cell-mediated immune response, thus reducing the ability of the body to clear the virus. Several previous studies have reported that corticosteroid therapy is linked to persistent viral RNA shedding in patients with MERS and SARS [40, 41]. Xu et al also showed that corticosteroid use was related to prolonged viral shedding, but this was not found to be an independent risk factor in the multivariable model [16]. Nevertheless, our results showed that systemic glucocorticoid therapy was independently associated with prolonged duration of viral shedding in patients with COVID-19 regardless of disease severity. In addition, among the non-severe COVID-19 patients, female individuals and patients who had received lopinavir/ritonavir treatment early showed shorter duration of viral shedding. Recent reports showed that early antiviral treatment could shorten SARS-CoV-2 viral shedding duration in patients with COVID-19 [42, 43]. Our results further confirmed the significance of early antiviral treatment only in non-severe patients, and a recent randomized controlled trial also indicated that lopinavir-ritonavir treatment was not associated with clinical benefits, including viral shedding, in patients with severe COVID-19 [44]. Nevertheless, further clinical trials on the effects of early administration of lopinavir-ritonavir in patients with non-severe COVID-19 remain needed. The sex-related difference in duration of viral RNA shedding was consistent with the findings of a previous report, and this phenomenon may be explained by sex-specific immune responses related to sex hormones or ACE2 expression [16]. Females are deemed to have stronger immune system responses than males, as they exhibit lower infection and mortality rates when faced with infectious diseases, and they also show higher responses to various vaccinations than do males [45]. Therefore, our findings have been added to the existing literature regarding viral RNA excretion patterns in patients with COVID-19 based on larger sample sizes.

There were several limitations to this study. First, our study was conducted using a retrospective data analysis. Second, given the variation in the clinical management among different hospitals, some cases had incomplete laboratory test results. All data generation was clinically driven and not systematic. Third, most patients had recovered and been discharged at the time of data analysis and only 3 patients died during admission, so the clinical factors associated with fatal outcomes and the difference of mortality rates in specific sub-populations could not be analyzed. Fourth, there was a limited number of hypertensive patients who received ACEI or ARB therapy, and we could not analyze the effect of AECI and ARB individually. A larger cohort study of COVID-19 patients with hypertension from other cities in China and other countries would help to further validate the potential effect of AECI/ARB therapy.

COVID-19 is a fast-evolving, global pandemic with a higher risk of severe or critical illnesses in individuals who are older, have underlying hypertension without receiving ACEI/ARB therapy, or have COPD. In addition, male gender, receiving lopinavir/ritonavir treatment early, and receiving systemic glucocorticoid therapy were independently associated with prolonged viral shedding. These findings may provide a rationale to clinicians for the risk stratification, medical resource allocation, and early intervention.

## Supplementary Materials

The Supplemenantry data can be found online at: www.aginganddisease.org/EN/10.14336/AD.2020.0630.
