# Supplementary material for: Clinical Factors Associated with Progression and Prolonged Viral Shedding in COVID-19 Patients: A Multicenter Study
Source: Aging Dis. 2020 Oct 1;11(5):1069–81. doi: 10.14336/AD.2020.0630 (PMC7505267; doi:10.14336/AD.2020.0630)
Supplement: Supplementary file 1 — The Supplemenantry data can be found online at: www.aginganddisease.org/EN/10.14336/AD.2020.0630. [file AD-11-5-1069-suppl.pdf]

SUPPLEMENTARY DATA

Clinical Factors Associated with Progression and Prolonged Viral Shedding in COVID-19 Patients: A Multicenter Study

Zhichao Feng[1, 2], Jennifer Li[3], Shanhu Yao[1, 2], Qizhi Yu[4], Wenming Zhou[5], Xiaowen Mao[6], Huiling Li[1], Wendi Kang[1], Xin Ouyang[7], Ji Mei[8], Qiuhua Zeng[9], Jincai Liu[10], Xiaoqian Ma[1, 2], Pengfei Rong[1, 2, *], Wei Wang[1, 2,*]

© 2020. Feng Z et al. Published online at http://www.aginganddisease.org/EN/10.14336/AD.2020.0630

SUPPLEMENTARY DATA

Supplementary Table 1. Clinical risk factors associated with development of severe or critical illnesses according to age.

Variables	Age ⩾ 50 years		Age < 50 years	
	Multivariate OR (95% CI)	*P* value	Multivariate OR (95% CI)	*P* value
Male gender		0.754		0.209
Smoking history		0.083		0.250
Hypertension		**0.004**		0.866
Non-hypertensive	Reference	1		1
Hypertensive on AECI/ARB therapy	0.49 (0.06-3.96)	0.504		0.688
Hypertensive on other therapy	3.16 (1.54-6.48)	**0.002**		0.717
Diabetes		0.359		0.469
Cardiovascular disease		0.647		0.603
COPD	8.37 (2.75-25.48)	**< 0.001**		—
Obesity		0.143		0.637

Abbreviations: ACEI, angiotensin-converting enzyme inhibitors; ARB, angiotensin-receptor blockers; CI, confidence interval; COPD, chronic obstructive pulmonary disease; OR, odds ratio.

Supplementary Table 2. Clinical risk factors associated with development of severe or critical illnesses according to gender.

Variables	Male		Female	
	Multivariate OR (95% CI)	*P* value	Multivariate OR (95% CI)	*P* value
Age	1.04 (1.01-1.07)	**0.010**	1.05 (1.02-1.09)	**0.001**
Smoking history		0.398		0.464
Hypertension		0.504		0.099
Non-hypertensive		1	Reference	1
Hypertensive on AECI/ARB therapy		0.999	1.90 (0.20-18.04)	0.578
Hypertensive on other therapy		0.241	2.83 (1.09-7.37)	**0.033**
Diabetes		0.523		0.122
Cardiovascular disease		0.660		0.641
COPD	10.84 (2.52-46.637)	**0.001**		0.579
Obesity		0.354		0.057

Abbreviations: ACEI, angiotensin-converting enzyme inhibitors; ARB, angiotensin-receptor blockers; CI, confidence interval; COPD, chronic obstructive pulmonary disease; OR, odds ratio.

© 2020. Feng Z et al. Published online at http://www.aginganddisease.org/EN/10.14336/AD.2020.0630
